# Ameliorating Effects of Auricular Electroacupuncture on Rectal Distention-Induced Gastric Dysrhythmias in Rats

**DOI:** 10.1371/journal.pone.0114226

**Published:** 2015-02-02

**Authors:** Zhaohui Zhang, Jieyun Yin, Jiande D. Z. Chen

**Affiliations:** 1 Department of Acupuncture, the First Affiliated Hospital, Nanjing Medical University, Nanjing, China; 2 Division of Gastroenterology, University of Texas Medical Branch, Galveston, Texas, United States; 3 Ningbo Pace Translational Medical Research Center, Beilun, Ningbo, China; University of Texas Medical Branch, UNITED STATES

## Abstract

Gastric slow waves (GSW) are known to regulate gastric motility and are impaired with rectal distention (RD). Electroacupuncture (EA) at body acupoints, such as ST 36, has been shown to improve gastric dysrhythmias; however, little is known about the possible effects of auricular electroacupuncture (AEA) on GSW. To study effects and possible mechanisms of AEA on RD-induced gastric dysrhythmias in rats, ten male Sprague-Dawley (SD) rats implanted with gastric serosal electrodes were studied in two different experiments in fed state. Four sessions were performed in experiment 1 as follows: control (RD, no stimulation), RD+AEA, RD+EA at body points and RD+sham AEA. Two sessions were included in experiment 2 to study mechanisms of AEA: RD + atropine and RD + atropine + AEA. It was found that 1) RD significantly decreased the percentage of normal GSW from 89.8±3.5% to 76.0±3.3% (*P*<0.05); 2) AEA increased the percentage of normal GSW during RD to 94.0±2.1% (*P*<0.05 vs. RD) via a reduction in the percentages of tachygastria and arrhythmia (*P*<0.05 vs. RD); 3) atropine blocked the ameliorating effect of AEA on RD-induced gastric dysrhythmias. Our results demonstrated that RD induces gastric dysrhythmias in fed state in rats. AEA improves RD-induced gastric dysrhythmias via the vagal pathway. AEA may have a therapeutic potential in treating gastric dysrhythmias.

## Introduction

It is well known that myogenic, neuronal and hormonal factors are very important to normal gastric motility [1, 2]. However, normal gastric motility ultimately depends on the depolarization and repolarization of gastric smooth muscles, causing contractions and relaxations of the muscle while interstitial cells of Cajal (ICC), extensively distributed in the muscular wall of the gastrointestinal tract, plays an important role as the pacemaker to generate slow waves spontaneously [2]. Gastric slow waves (GSW) can be recorded internally via electrodes implanted on gastric serosa [3] or noninvasively via abdominal surface electrodes, a method called Electrogastrography (EGG) [4–6]. Gastric dysrhythmias have been associated with abnormal gastric emptying [7, 8] abnormal gastric motility [9, 10] and unexplained nausea and vomiting [11, 12]. Rectal distention (RD), a physical stimulation, has been proven as a good way to induce gastric hypomotility, and delayed gastric emptying (GE) with concurrent gastric dysrhythmias in both animals [13] and humans [14]. Thus RD serves as an excellent non-pharmacological model of gastric dysrhythmias.

Auricular acupuncture (AA), a diagnostic and treatment system derived from ancient China, Egypt and Greece, is now widely used in clinic through stimulation of points on the auricles [15]. It has been reported that a number of functional gastrointestinal diseases, such as functional dyspepsia (FD) [16] and constipation [17], might be treated with AA. AA could effectively decrease the incidence and degree of cisplatin-induced delayed nausea and vomiting [18]. The acid production could be suppressed in gastric body and alkalinizing function of the antrum could be improved as well when AA therapy was combined with body acupuncture therapy [19]. Gastric peristalsis time could be prolonged with acupressure at auricular points [20]. It is well known that gastric motility is regulated by certain gut hormones [21] and neurotransmitters [22] through vagal afferents. The vagus nerves play a crucial role in regulating gastric motility. Activation of vagal nerves is known to enhance gastric motility. AA was recently reported to improve delayed gastric emptying induced by anhydrous ethanol in rats mediated via the vagal pathway [23]. Auricular electroacupuncture (AEA) is a method in which weak electrical stimulation is performed via fine acupuncture needles inserted into acupoints in the auricula. It has been reported to treat obesity and depression in clinic [24, 25]. However, little is known whether AEA is effective in treating gastric dysfunctions.

EA at ST36 has been reported to improve impaired gastric motility and gastric dysrhythmia in dogs [26] and rats [27] via the vagal mechanism. We speculated that AEA would also improve gastric dysrhythmias via the vagal pathways. The aim of this study was to investigate the effects of AEA and EA on RD-induced gastric dysrhythmias in rats and to expose the possible mechanisms especially the vagal pathways of AEA involved in improving gastric dysrhythmias induced by RD in rats.

## Materials and Methods

### Ethics statement

All animal procedures performed in this work were approved by the Animal Care and Use Committee of the University of Texas Medical Branch at Galveston, Texas.

### Animal preparation

Ten adult male Sprague_Dawley (SD) rats, weighting 250g–300g (Harlan Laboratories, Houston, TX, USA), were housed in individual plastic cages. All cages with experimental animals were in rooms with temperature of 22°C, humidity of 40% and a 12:12 h light/dark cycle. Water and food were available ad libitum. Experimental rats were acclimatized to the new circumstance within 1 week after arrival.

### Surgical Procedure

One week before the experiment, ten rats were implanted with serosal electrodes for recording GSW described as follows: After an overnight fast, the rat were operated under anesthesia with the inhalation of 1.5–2.0% isoflurane (Terrell, Piramal Critical Care, Inc. USA). A midline laparotomy was performed, then one pair of 28-gauge cardiac pacing wires (A&E Medical, Farmingdale, NJ, USA) were implanted on the gastric serosal surface about 0.5 cm proximal to the pylorus with an inter-electrode distance of 0.5cm. Electrode connecting wires were tunneled subcutaneously through the anterior abdominal wall and externalized at the middle neck back. After the surgical procedure, the animals were given 7 days to recover before the experiment.

### AEA, Sham-AEA and EA

Two stainless steel needles (Model Hua-tuo 0.5 mm diameter, Medical Appliance Factory Co., Ltd, Suzhou, China) were inserted bilaterally at the stomach point in the auricles of the rat and then punched through the auricular gristle for about 3mm and curved the pinpoints at the narcotized state. The stomach point was located between the cymba conchae and cavum conchae [28]. Electrical stimulation was performed via the needles using a universal pulse generator (Pulsemaster A300 and Stimulus Isolator, World Precision Instruments, USA). Stimulation parameters were set as follows: pulse trains with a 2s on time and a 3s off time; pulse frequency of 25Hz and amplitude of 0.7mA. This set of parameters was used in EA studies successfully in improving gastric motility in our lab [26, 27]. The electrical stimulation was delivered for the entire 30 min of RD. Sham-AEA was performed using the same parameters but via needles inserted at bilateral earlobes. EA stimulation was performed on the ST-36 points. ST36 point in the rat was located at 5mm below head of fibula under knee joint, and 2mm lateral to the anterior tubercle of the tibia [27]. A pair of suture needles was inserted bilaterally at a depth of 3–5 mm into the skin at ST36. The ST36 was electrically stimulated using the same pulse generator and same stimulation parameters as AEA except for the amplitude of 1.5mA.

### Rectal Distention (RD)

Under general anesthesia with the inhalation of isoflurane, a flexible balloon (5cm) constructed from a surgical glove finger attached to a Tygon tubing was inserted 8 cm into the descending colon and rectum via the anus and held in place by taping the tubing to the tail. The rat was placed in a restrainer and allowed to adapt for 30 min. The balloon was then inflated with air to a degree such that the animal felt distention but was able to tolerate it. The average distension level was 45 mmHg. The distention pressure was measured by the mercury sphygmomanometer and held constant through the RD period in each session. There was a slight variation in distention pressure among individual rats. However, the distention pressure used in different sessions for each specific animal was fixed at the same level.

### Experiment Protocols

**Figure 1:**
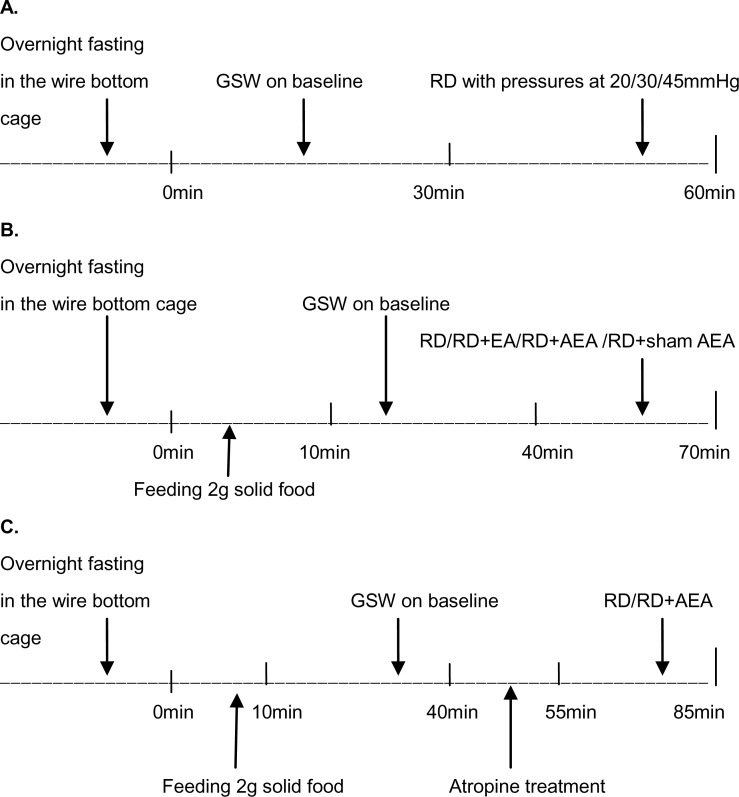
Experimental protocol. (A) GSW recordings in the fasting state at baseline and during RD; (B) GSW recordings in the fed state before and during RD or RD plus EA/AEA/sham-AEA; (C) GSW recordings in the fed state before RD and during RD at presence of atropine.

**Experiment 1**. This experiment was designed to study the effect of EA/AEA on RD-induced gastric dysrhythmias and conducted in three fasting sessions and 4 randomized fed sessions on separate days described as follows. The fasting sessions were performed to study the effects of RD at different pressures on GSW in the fasting state. These fasting sessions were performed one week after surgery on three different days. GSW were recorded in the 10 overnight fasted rats for 30 min as baseline and then for 30 min with RD. Three different RD pressures (20, 30 and 45mmHg) were used, respectively on three days. There was 2-day intermission between two tests (Fig. 1A).

The postprandial sessions included RD only, RD+AEA, RD+sham-AEA and RD+EA at ST36. Fig. 1B illustrates the protocol of these experimental sessions. In brief, the animals were fasted overnight and fed with 2g of regular solid food. After feeding, GSW were recorded for 30 min without RD and 30 min with RD with or without EA/AEA/sham-AEA. There was a recovery period of at least 2 days between two consecutive sessions.

**Experiment 2**. Two sessions were included in experiment 2 to study the vagal mechanism involved in the effects of AEA on RD-induced abnormal GSW. The experiment was performed on two different days with an interval of at least 2 days in a randomized order: RD+atropine and RD+atropine+AEA (Fig. 1C). The same 10 rats were fasted overnight and then fed with 2g of solid food within 10mint. GSW were recorded for 30 min at baseline before RD and 30 min during RD or RD with AEA. Atropine (2.5 mg kg^-1^, American Regent, Inc, NY, USA) was injected subcutaneously after the baseline recording and 15 min before RD.

### Recording and Analysis of GSW

GSW were recorded by a multi-channel recorder (Acknowledge, UIM100A; Biopac Systems, Santa Barbara, CA) as described in previous studies [27, 29]. The GSW signals were displayed and saved on a personal computer. The parameters of GSW, including the percentage of normal GSW, the percentage of bradygastria/ tachygastria/arrhythmia, dominant frequency (DF), and dominant power (DP), were calculated and analyzed using a previously validated method [30].

### Statistical analysis

All data are presented as means±SE. Analysis of variance (ANOVA) was used to investigate the difference in each of GSW parameters among different sessions. Student’s t-test was used to compare the difference in each parameter between the baseline and during RD in each session. Statistical significance was assigned at *P* <0.05.

## Results

### Effects of RD on GSW

**Figure 2:**
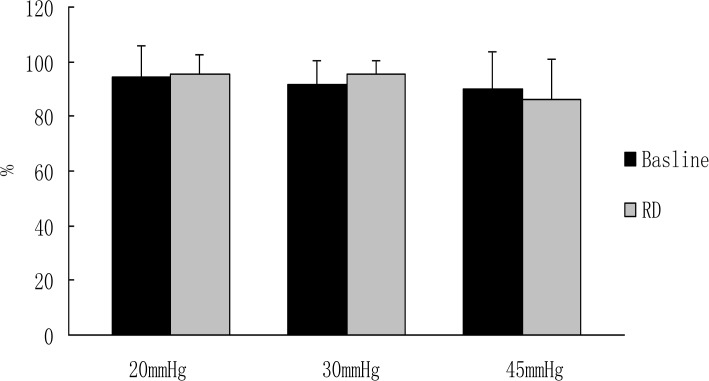
Effects of RD on GSW at different pressures in fasting state. The percentage of normal GSW was not changed significantly (P>0.05) after RD with different pressure.

In the fasting state, RD did not induce any significant change in the percentage of normal GSW in rats at different pressures (Baseline: 94.6±11.6% at 20mmHg session, 91.8±8.78% at 30mmHg session, 90.0±13.7% at 45mmHg session vs. those during RD: 95.5±7.2% at 20mmHg, 95.5±5.0% 30mmHg, 86.1±14.7% at 45mmHg during RD, *P*>0.05) (Fig. 2).

**Figure 3:**
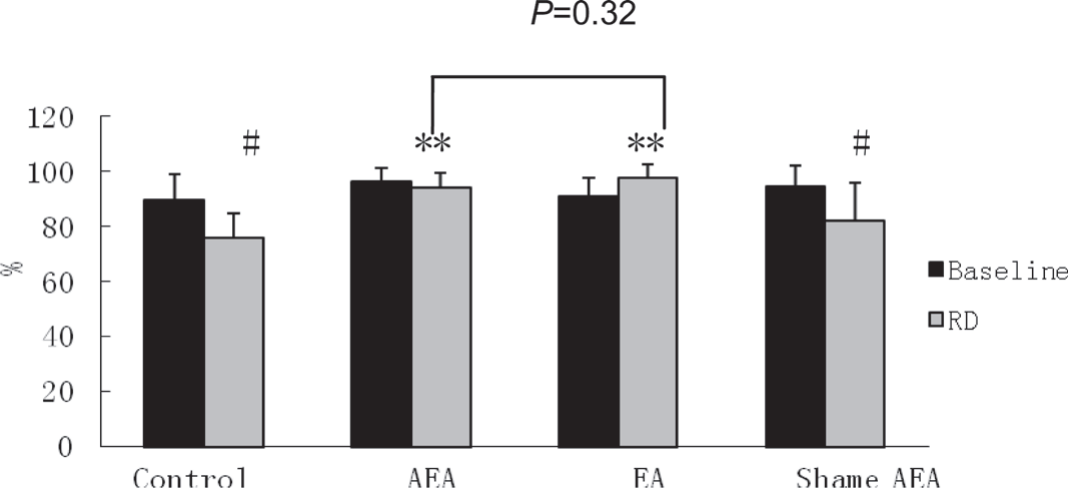
Effects of EA and AEA on RD-induced abnormal GSW. The percentage of GSW decreased significantly after RD in Control and Sham AEA group (^#^P<0.05 RD vs baseline). While the abnormal GSW induced by RD were repaired significantly after using EA or AEA with RD (**P<0.05 RD plus EA/AEA vs RD). The effect between EA and AEA on repairing the impaired GSW induced by RD was no significant difference (P = 0.32).

**Figure 4:**
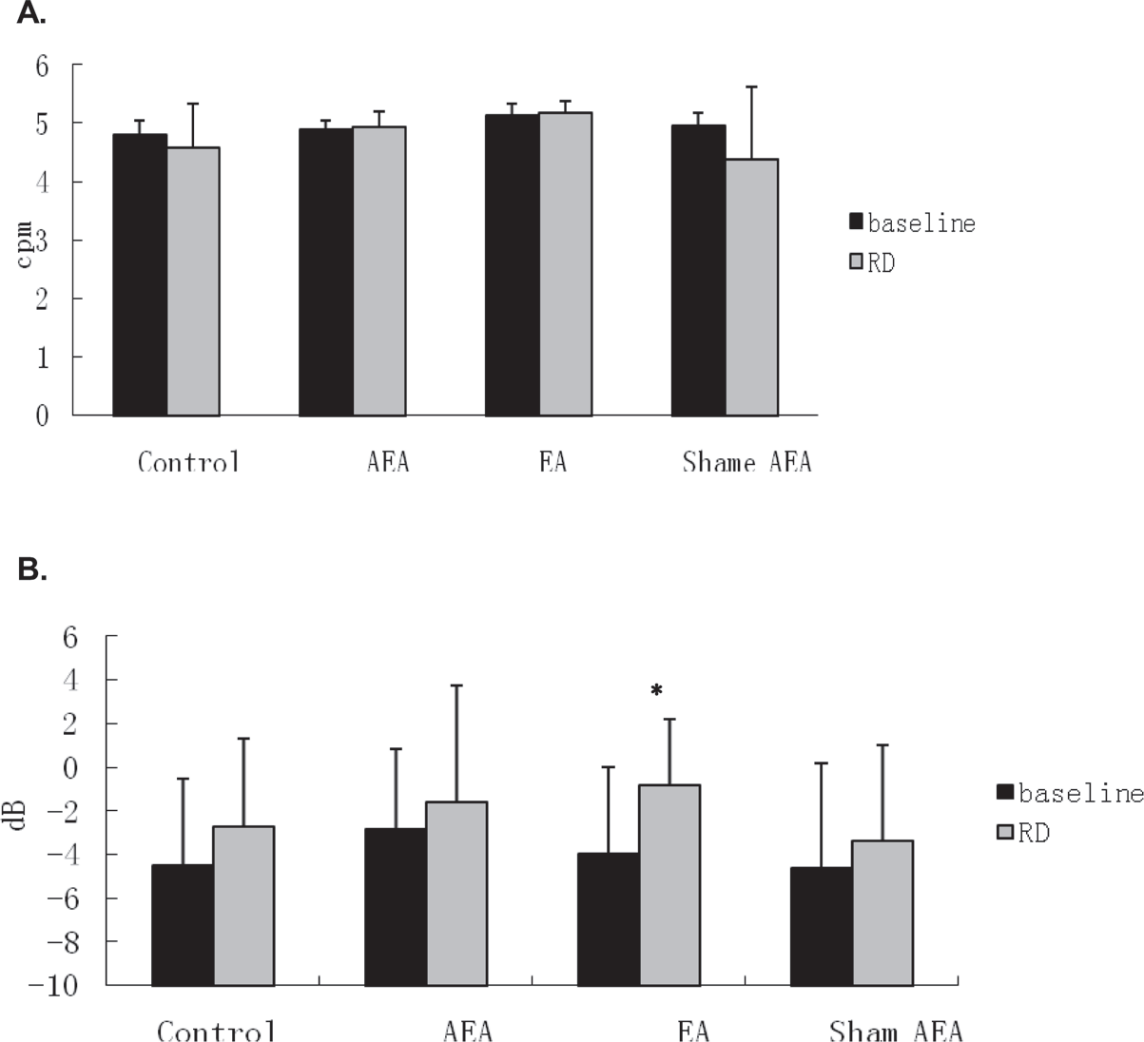
Effects of RD on Dominant frequency (DF) and dominant power (DP) of GSW. (A) DF was not altered significantly (P>0.05) after treatment comparing with that of baseline in each session. (B) EA at ST-36 increased DP of GSW significantly (*P<0.05, vs. baseline).

**Figure 5:**
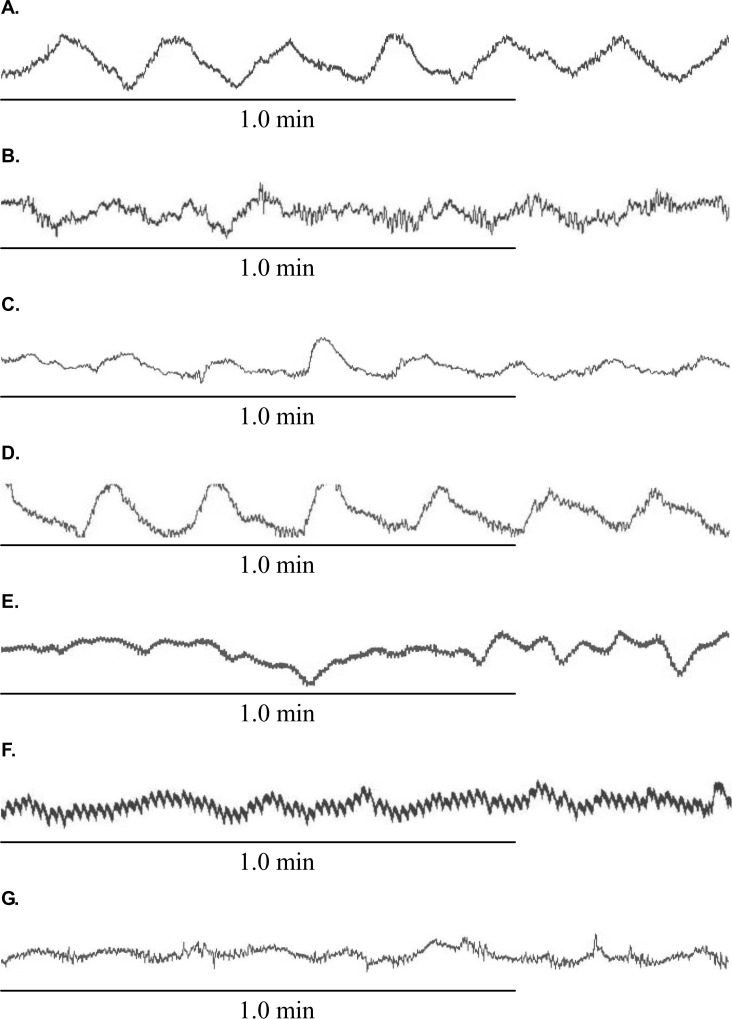
Typical GSW tracings in the fed state at different sessions. (A) GSW on baseline. (B) GSW at RD session. (C) GSW at RD+AEA session. (D) GSW at RD+EA session. (E) GSW at RD+sham AEA session. (F) GSW at RD+atropine session. (G) GSW at RD+atropine+AEA session.

In the fed state, however, the percentage of normal GSW was decreased significantly during RD: 89.8±3.5% without RD vs. 76.0±3.3% with RD at 45 mmHg, (*P*<0.05). However, RD did not significantly alter DF (4.8±0.1 dB without RD vs. 4.6±0.3 dB with RD, *P*>0.05) or DP (-4.5±1.5 dB without RD vs. -2.7±1.5 dB with RD, *P*>0.05) (Figs. 3 and 4). As shown in Fig. 5(A and B) a substantial distortion was noted during RD.

### Effects of AEA and EA on RD-induced impairment in GSW

AEA and EA improved the impaired GSW induced by RD. As shown in Fig. 5, the GSW tracing during RD became more regular after AEA (tracing C) and EA (tracing D) but not after sham-AEA stimulation (tracing E) in comparison with that during RD alone (tracing B). In the RD plus AEA session, the percentage of normal GSW remained unchanged during RD (94.0±2.1%), compared with the baseline before RD (96.5±1.8%, *P*>0.05) (Fig. 3). This value was significantly higher than that in the corresponding period (during RD) in the RD session without AEA (*P*<0.05). Similar to the RD session, RD did not alter DF or DP in the RD plus AEA session (Figs. 3 and 4).

The effects EA at ST-36 on GSW were similar to AEA. In the RD plus EA at ST36 session, the percentage of normal GSW was not altered by RD (Baseline 91.3±2.5% vs. 97.6±1.9%, *P*>0.05), demonstrating a preventive effect of EA at ST-36 on RD-induced impairment in GSW (Fig. 3), Furthermore, there was no significant difference in the percentage of normal GSW between the RD plus AEA session (94.0±2.1%) and RD plus EA at ST36 session (97.6±1.9%) during RD (*P*>0.05) (Fig. 3), suggesting that these two methods of electroacupuncture were equally effective in preventing RD-induced GSW impairment. Interestingly, however, EA at ST-36 was noted to increase DP during RD; in the RD plus EA session, DP increased significantly from -3.9±1.5 dB at baseline to -0.8±1.1 dB during RD and EA (P<0.05) (Fig. 4), indicating that EA also enhanced slow wave intensity, which was not the case with AEA.

**Figure 6:**
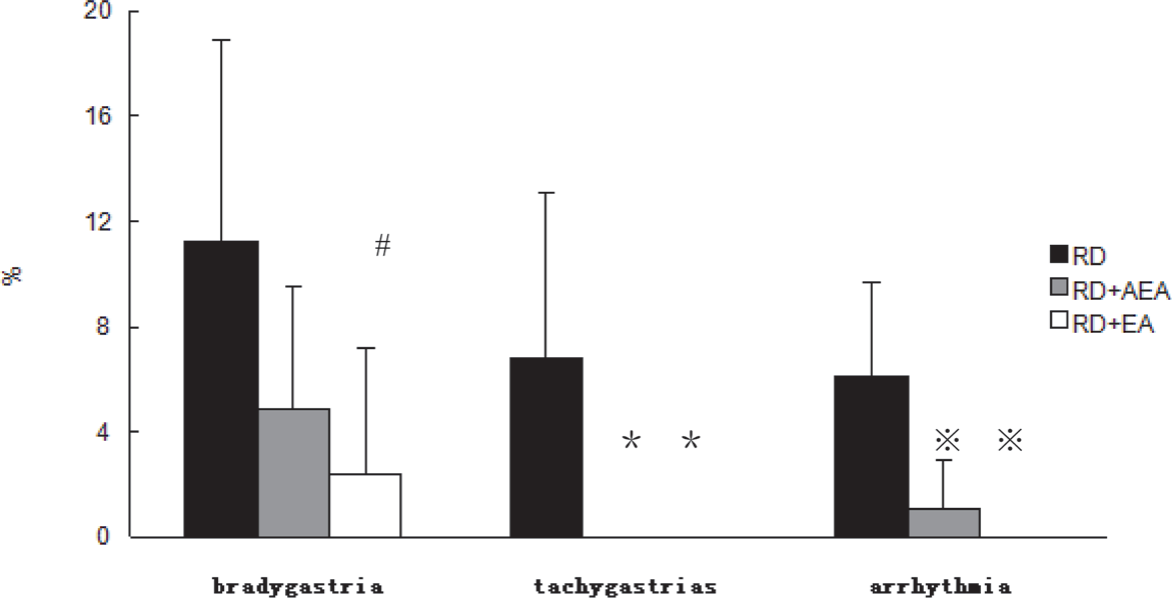
Effects of AEA on RD-induced gastric dysrhythmia. EA reduced the percentage of bradygastria (B%) significantly during RD (#P<0.05, vs. RD). The percentage of tachygastrias (T%) decreased to 0% after using EA and AEA respectively during RD (^＊^P<0.05, vs. RD). The percentage of arrhythmia (A%) decreased significantly as well after using EA and AEA respectively during RD (^※^P<0.05, vs. RD).

The improvement in RD-induced impairment in GSW with AEA and EA was found to be attributed to the improvement (reduction) in gastric dysrhythmias reflected as follows. 1) The percentage of bradygastrias (B%) induced by RD was decreased significantly with EA at ST-36 (11.2±7.7% during RD alone vs. 2.4±4.8% during RD and EA, *P*<0.05), but not with AEA (4.9±4.6%, *P*>0.05 vs. RD alone). 2) The percentage of tachygastrias (T%) induced by RD was decreased significantly after both EA and AEA (6.8±6.3% during RD alone vs. 0.0±0.0% during RD plus AEA or EA, *P*<0.05). 3) The percentage of arrhythmia (A%) induced by RD was decreased significantly as well after both EA and AEA: 6.1±3.6% during RD, 1.1±1.6% during RD plus AEA (*P*<0.01) and 0.0±0.0% during RD plus EA (*P*<0.01) (Fig. 6).

### Atropine impaired GSW

**Figure 7:**
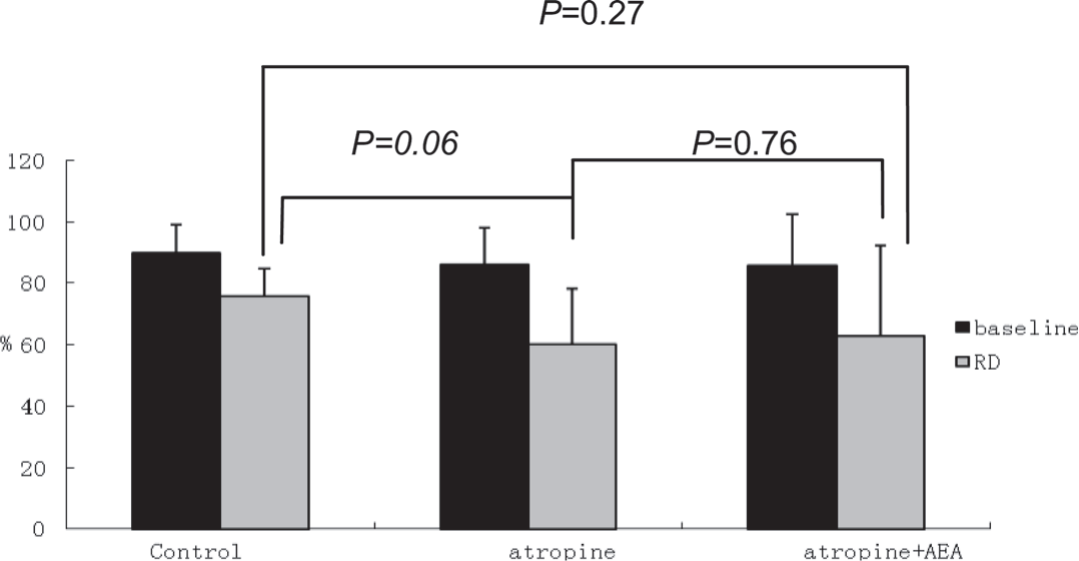
Atropine blocked the preventive effect of AEA on RD-induced impairment in GSW. There was no significant difference of the percentage of GSW among 3 sessions after RD, RD+atropine or RD+atropine+AEA respectively (P>0.05).

In the RD plus atropine session (fed state), the percentage of normal GSW was decreased significantly from 86.0±4.3% at baseline to 60.3±6.3% during RD (*P*<0.05) that was lower than the corresponding value in the RD alone session (76.0±3.3%) although the difference was not statistically significance (*P* = 0.06) (Fig. 7 and Fig. 5F). Atropine worsened the inhibitory effects of RD on slow wave frequency and power. At the presence of atropine, RD significantly increased DF (4.80±0.07 cpm at baseline and 5.80±0.24 during RD, *P*<0.05) and reduced DP (-3.1±2.4 dB at baseline vs. -10.2±1.8 dB during RD, *P*<0.05) (Table 1).

**Table 1:** Effects of atropine treatment on the DF and DP of GSW.

Group	Session	DF(cycles/minute)	DP(dB)
Control	Baseline	4.8±0.09	-4.5±1.5
	RD	4.6±0.29	-2.7±1.5
Atropine	Baseline	4.8±0.07	-3.1±2.4
	RD plus atropine	5.8±0.24*	-10.2±1.8*
Atropine plus AEA	Baseline	4.3±0.45	-2.8±2.6
	RD plus atropine plus AEA	5.2±0.47	-7.0±1.6

* *P*<0.05 vs. baseline.

### Atropine blocked the effect of AEA

At the presence of atropine, AEA was incapable of normalizing or preventing RD-induced impairment in GSW. As shown in Fig. 7, no difference was noted in the percentage of normal GSW during RD among three sessions of RD alone, RD plus atropine, and RD plus atropine plus AEA. Similarly, at the presence of atropine, AEA was not able to alter DF or DP of GSW during RD, in comparison with the control session (RD alone) or RD plus atropine session (Table 1, Fig. 5G).

## Discussion

The present study demonstrated followings: 1) RD could impair rodent GSW in the postprandial but not fasting state; 2) similar to EA at ST36, AEA was able to improve and actually normalize or prevent RD-induced impairment in postprandial GSW; 3) the ameliorating effect of AEA on RD-induced gastric dysrhythmia was mediated via the vagal pathway.

While EA at ST36 has been commonly used for treating functional gastrointestinal disorders, it is unknown whether auricular EA (AEA) has similar effects on gastrointestinal motility. To answer this question, we designed a study to investigate possible effects of AEA on gastric dysrhythmia induced by RD in comparison with EA at ST36. The gastric slow wave is known to regulate gastric motility, such as determining the frequency and propagation of gastric contractions. It can be easily and reliably measured in rats, and can be altered with rectal distention. Accordingly, we chose the gastric slow wave as a surrogate measure of gastric contractions in this study. In choosing stimulation point for AEA, we picked up the stomach point in the ear that is located at the edge of auricular concha distributing the branch of vagus abundantly [31, 32]. The earlobe was selected as the sham point since it is not innervated with vagal nerves [33]. The selection of stimulation parameters for AEA was based on previous studies with EA at ST36 [26, 27, 34–36] except that a lower stimulation intensity was used for AEA than EA since the animal was more sensitive to ear stimulation than stimulation at ST36.

RD was used to impair gastric slow waves. Previously, RD was reported to impair gastric slow waves only in the postprandial state but not fasting state in humans [37, 38]. In dogs, however, gastric slow waves were impaired with RD in both fasting and fed states [13, 26]. In rats, the current study indicated that gastric slow waves were not altered by RD even at a pressure of 45mmHg or below in the fasting state but were impaired in the fed state at an average pressure of 45 mmHg. These findings suggest that gastric slow waves are more vulnerable to exogenous interferences in the postprandial state than the fasting state. Although not measured in the current study, previous experiments in dogs demonstrated a concurrent inhibition of antral contractions [26] and delayed gastric emptying [39] during rectal distention. Further more, the inhibitory effects of RD on gastric slow waves and antral contractions were mediated via the sympathetic activation [26].

While numerous studies have shown the ameliorating effects of EA at ST36 on gastric slow waves and motility, to the best of our knowledge, this was the first study demonstrating a similar ameliorating effect of auricular EA on gastric slow waves. In dogs, EA at ST36 was reported to improve gastric dysrhythmias and antral hypomotility as well as delayed gastric emptying induced by RD [26, 39]. In humans, acupuncture (AP) at ST36 was found to improve gastric dysrhythmias and abdominal symptoms induced by RD [38]. In this study, as expected, EA at ST36 normalized or prevented RD-induced gastric dysrhythmias. Interestingly, AEA showed a similar normalizing or preventive effect on RD-induced gastric dysrhythmias. The percentage of normal GSW during RD was 94% that was almost the same as that before RD (96%). Further analysis revealed complete elimination of tachygastria and almost complete elimination of arrhythmia with AEA during RD.

The ameliorating effect of AEA on RD-induced gastric dysrhythmias is believed to be mediated via the vagal mechanism. Anatomically, the gastric point in the ear is innervated with Arnold nerve, a branch of the vagus [40]. It was shown that needling gastric points in auricular of rats caused the expression of neuropeptide Y in the hypothalamus increased significantly in the fed state [28] and some feeding-related hypothalamic neuronal activities were modulated significantly after stimulating the cavum conchae areas in auriculars of obese rats with low frequency electroacupuncture [41]. In the current study, the effect of AEA on gastric dysrhythmias was completely blocked by atropine, a muscarinic receptor antagonist [42, 43] known to block vagal nerve activity [44, 45]. These findings suggested a vago-vagal mechanism involved in the ameliorating effect of AEA on RD-induced gastric dysrhythmia; electrical stimulation at the gastric point in the ear activated the vagal nerve that led to a central response in the brain stem, such as dorsal motor nucleus of the vagus [46, 47] or in the hypothalamus [28, 41], and the activation of the central nerves by AEA solicited an enhanced vagal efferent activity to the stomach, resulting in improvement in gastric slow waves. The similar vagal mechanism was also consistently reported with EA at ST36 in our previous studies. In both humans and dogs, RD induced gastric dysrhythmias and reduced vagal activity measured by the spectral analysis of the heart rate variability; EA in dogs or acupuncture in humans at ST36 increased vagal activity and improved gastric slow waves during RD [26, 38]. In diabetic rats, EA at ST36 was also reported to improve gastric motility of model rats via vagal pathways [27].

The other two major parameters, dominant frequency and dominant power of gastric slow wave recordings were not significantly altered by RD. It should be pointed out. However, these two parameters reflect the averages of the entire recording, unlike the percentage of normal slow waves or percentage of tachygastria/ bradygastria/ arrhythmia that reflect minute-by-minute changes in slow wave rhythmicity [30]. It is conceivable that the overall parameters were less sensitive to intervention.

It is well known that ST36 and PC6 are good acupoints in regulating gastrointestinal functions not only in humans but also in animals [48]. However, up to now, there has not been any report comparing the long term effects of AEA and body electroacupuncture at ST36 or P6 on gastrointestinal motility in humans. Further studies are needed to determine whether AEA is better than EA at ST36 or PC6, or vice versa.

## Conclusion

In summary, Auricular EA at the gastric point normalizes RD-induced gastric dysrhythmias and its effect is mediated via the vagal mechanism. Similar to EA at body points, such as ST36, auricular EA may also have therapeutic potential for treating gastric motility disorders. Further clinical studies are warranted.
